# 

**Published:** 1817-03

**Authors:** 


### CORRESPONDENCE.

Dr. Mateos' Remarks on the degraded State of Medicine in Spain will appear in our next.

Reflections on the Limits between Physic and Surgery are received.

One of the Editors purposes to answer, in a short time, the queries of "*A young but constant Reader.*" Our juvenile Correspondent, however, should be aware, that a subject of such delicacy and importance is not to be lightly taken up, or discussed without deep and deliberate reflection. In the interim, we would recommend him to employ at least one hour daily, *early in the morning*, in acquiring or improving an already acquired knowledge of the Latin and French languages; and two hours at night in reading, and *digesting*, the last edition of Dr. Duncan's valuable Dispensatory.

The Transactions of the Medical Society of London, Vol. I. Part II. and Dr. Crawford's Work on the Effects of Tonics are received, and will be early reviewed.

☞ Authors and Publishers are requested to send Copies of new Works for Review as early as possible after their publication. They will be reviewed early in proportion.

